# High-risk features of basilar artery atherosclerotic plaque

**DOI:** 10.3389/fneur.2022.1019036

**Published:** 2022-10-28

**Authors:** Shaojun Li, Jiana Wei, Ruiyun Huang, Chenghao Li, Hongbing Chen, Zhihua Qiu, Yongjun Jiang, Li Wu

**Affiliations:** ^1^Department of Neurology, The Second Affiliated Hospital of Guangzhou Medical University, Guangzhou, China; ^2^Department of Neurology and Stroke Center, The First Affiliated Hospital, Sun Yat-sen University, Guangzhou, China

**Keywords:** intraplaque hemorrhage, ruptured fibrous cap, high-resolution MRI, basilar artery stenosis, atherosclerotic plaque

## Abstract

**Introduction:**

High-resolution magnetic resonance imaging (HR-MRI) is used to characterize atherosclerotic plaque. The present study aimed to determine the high-risk features of the basilar artery (BA) atherosclerotic plaque.

**Methods:**

Patients with advanced BA stenosis were screened. The features including the ruptured fibrous cap (RFC), lipid core, intraplaque hemorrhage (IPH), plaque enhancement, and calcification were assessed by using high-resolution MRI. The relationship between the features and acute infarction was analyzed.

**Results:**

From 1 June 2014 to 31 December 2018, a total of 143 patients with 76 new strokes were included. RFC was identified in 25% of symptomatic and 10.4% of asymptomatic patients. IPH was identified in 48.7% of symptomatic and 25.4% of asymptomatic patients. RFC (3.157, 95% CI 1.062 to 9.382, *p* = 0.039) and IPH (2.78, 95% CI 1.127 to 6.505, *p* = 0.026) were independent risk factors for acute infarction.

**Conclusion:**

Our study showed that RFC and IPH of BA plaque were independent risk factors for acute infarction.

## Introduction

Basilar artery (BA) atherosclerotic plaque is the predominant cause of stroke in the posterior circulation, which contributes to about 20% of all ischemic strokes (1). The underlying mechanism is that platelets adhere to the plaque surface to form the thrombus, which could block the upstream blood flow to induce a new stroke (2). In recent decades, advanced luminal stenosis was recognized as the main risk for developing stroke. However, the degree of stenosis guided intervention failed in the SAMMPRIS trial, which recruited 100 patients with significant BA stenosis (3). Therefore, the clinical significance of atherosclerotic plaque was shifted from the degree of stenosis to the features of plaque (4). The features of vulnerable plaque included a thin/ruptured fibrous cap (RFC) overlaying a large necrotic lipid core, intraplaque hemorrhage (IPH), plaque enhancement, and calcification (2, 5).

High-resolution magnetic resonance imaging (HR-MRI) is a non-invasive imaging modality to characterize plaque *in vivo* (6). Previous studies investigated the clinical significance of IPH in the BA plaque while the results remained inconsistent. Yu et al. found that 42.3% of BA plaques had IPH, which was a marker of the culprit lesion (7). Wang's study found no relationship between IPH in the BA plaque and the onset of stroke (8). As for the other features, studies involving HR-MRI are scarce.

The present study aimed to investigate the clinical significance of features of BA plaque in acute stroke.

## Methods

### Patient profile

The research protocol was reviewed and approved by the ethics committees of each institute according to the principles of the Declaration of Helsinki. Written consents were obtained from patients or their authorized relatives before enrollment.

Data were prospectively and consecutively collected from the participating institutions from 1 June 2014 to 31 December 2018. All enrolled patients underwent standard MRI scans to detect an acute stroke. Any neurological symptoms or signs in the posterior circulation, such as headache, dizziness, or vertigo, were screened by trained research coordinators at each participating center. HR-MRI scanning was performed in patients with significant BA stenosis (>50%) within 1 week after the initial MR examination. Patients were enrolled if they met the inclusion criteria as follows: (1) older than 18 years; (2) MRI [including T1-weighted imaging (T1WI), T2-weighted imaging (T2WI), and diffusion-weighted imaging (DWI)] and magnetic resonance angiography (MRA) were performed. (3) BA atherosclerotic plaque with 50–99% stenosis. (4) HR-MRI was successfully performed. Patients were excluded if they met any of the following criteria: (1) missing clinical or imaging information. (2) occlusion of BA. (3) non-atherosclerotic stenosis like dissection, aneurysm, moyamoya disease, or BA fenestration. (4) poor quality of HR-MRI images. (5) contraindications for gadolinium-based contrast agents. If intracranial vertebral arteries were terminated in the posterior inferior cerebellar artery, the origin of BA was considered at the medullary-pontine junction. A symptomatic case was defined as having a hyperintensity of DWI images located in the territory of the posterior circulation. Diagnosis of hypertension and diabetes were made according to the national guideline. The previous smoker was defined as those who had not smoked within the recent year. A current drinker was defined as having at least one drink within the recent year.

### MRI and HR-MRI scanning

All patients underwent MRI on admission using a 3.0T MRI unit (GE or Simens). Scanning sequences included T1WI, T2WI, T2-fluid-attenuated inversion recovery sequence, apparent diffusion coefficient maps, spectral presaturation with inversion recovery (SPIR), DWI, T2 black blood, and time-of-flight MRA (TOF-MRA) covering the circle of Willis.

The HR-MRI protocol consisted of pre- and post-contrast T1-weighted (repetition time [TR], 600 ms; echo time [TE], 12 ms; field of view [FOV], 12x12 cm; matrix size, 384x219; number of excitations [NEX], 4), T2-weighted (TR, 2910 ms; TE, 70 ms; FOV, 12x12 cm; matrix size, 384X269; NEX, 4), and proton density-weighted images (TR, 2910 ms; TE, 23ms; FOV, 12x12 cm; matrix size, 384x269; NEX, 4) with a 2-dimensional turbo spin echo sequence. The image review was conducted blindly by two senior neuroradiologists (CH and CL) in a core lab.

RFC was defined as partially invisible, surface irregularity on T1-weighted or T2-weighted images. IPH was defined as having signal intensity >150% of that of the adjacent gray matter in T1WI (7). Calcification was defined as having low signals on all pulse sequences. The lipid core was defined according to the previous report (9). Enhancement was compared using pre- and post-contrast 3D T1WI-SPACE images, where the signal intensity of the plaque was compared with the signal intensity of the pituitary (8).

### Statistics

The differences in continuous variables were compared using the homogeneity test of variances. Student's *t*-test was applied when normality assumptions were met; otherwise, the equivalent non-parametric test was used. The difference in categorical variables was compared with Pearson's Chi-square test. Cohen's kappa was used to determine the intra-observer agreement in the identification of the presence of RFC, lipid core, IPH, enhancement, and calcification. A univariate binary logistic regression analysis was performed for the effect of independent variables. Individual variables with a *p-*value < 0.2 during univariate analysis were selected for multivariate regression analysis, and the results were expressed as odd ratios (OR) and 95% confidence intervals (CI). *P* < 0.05 was considered statistically significant. SPSS 20.0 was used for statistical analysis.

## Results

**Figure 1:**
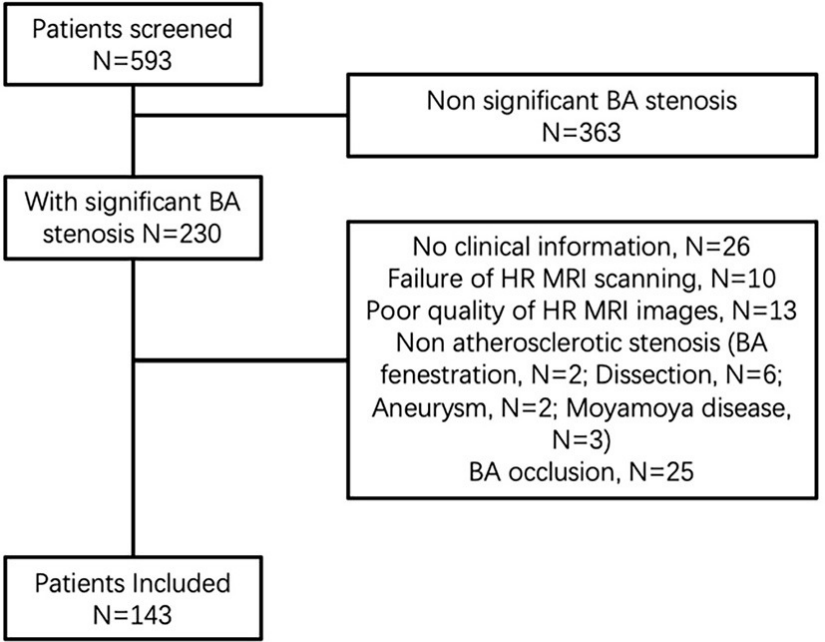
Flow diagram.

From 1 June 2014 to 31 December 2018, a total of 143 patients were finally enrolled (Figure 1). The baseline characteristics were summarized in Table 1. For the inter-reader agreement in assessment of RFC, lipid core, IPH, enhancement, and calcification, the kappa value was 0.900 (*P* = 0.049), 0.969 (*P* = 0.022), 0.953 (*P* = 0.027), 0.926 (*P* = 0.042), and 1.000 (*P* < 0.001), respectively. New stroke was found in 76 patients, and most of the stroke was located in the pons, medulla, and cerebellum (shown in Table 2).

**Table 1:** Baseline characteristics.

**Characteristics**	**Infarction**	**No infarction**	***P***
	***N* = 76**	***N* = 67**	
Age (yrs)	56.5 ± 11.7	57.2 ± 13.8	0.754
Male	57 (75.0%)	48 (71.6%)	0.650
Medical History			
Hypertension	42 (55.3%)	37 (55.2%)	0.995
New diagnosed	36 (47.4%)	32 (47.8%)	
Hypertension history	6 (7.9%)	5 (7.5%)	
Diabetes	24 (31.6%)	12 (17.9%)	0.164
New diagnosed	19 (25.0%)	10 (14.9%)	
Diabetes history	5 (6.6%)	2 (3.0%)	
AF	2 (2.6%)	1 (1.5%)	1.00
CHD	1 (1.3%)	1 (1.5%)	1.00
Previous stroke or TIA	8 (10.5%)	6 (9.0%)	0.752
Tumor history	4 (5.3%)	4 (6.0%)	0.854
Radiotherapy history	2 (2.6%)	1 (1.5%)	1.000
TC (mM)	4.23 ± 1.21	4.35 ± 1.18	0.574
LDL-C (mM)	2.89 ± 0.96	2.84 ± 1.09	0.881
HDL-C (mM)	1.03 ± 0.37	1.02 ± 0.25	0.786
TG (mM)	1.99 ± 1.06	1.86 ± 0.87	0.461
Smoker	30 (39.5%)	20 (29.9%)	0.445
Current smoker	24 (31.6%)	15 (22.4%)	
Previous smoker	6 (7.9%)	5 (7.5%)	
Drinker	15 (19.7%)	16 (23.9%)	0.700
Current drinker	13 (17.1%)	12 (17.9%)	
Previous drinker	2 (2.6%)	4 (6.0%)	
Medication			
Antihypertensive	35 (46.1%)	28 (41.8%)	0.608
Hypoglycemic	24 (31.6%)	11 (16.4%)	0.035
Statin	74 (97.4%)	59 (88.1%)	0.029
Antiplatelet	76 (100.0%)	67 (100.0%)	1.00
DAPT	49 (64.4%)	43 (64.2%)	
ASA or Clo	27 (35.5%)	24 (35.8%)	
Anticoagulation	1 (1.3%)	2 (3.0%)	0.600
Plaque characteristics			
Ruptured fibrous cap	19 (25.0%)	7 (10.4%)	0.024
Lipid core	26 (34.2%)	25 (37.3%)	0.699
IPH	37 (48.7%)	17 (25.4%)	0.004
Enhancement	11 (14.5%)	15 (22.4%)	0.221
Calcification	2 (2.6%)	6 (9.0%)	0.101

AF, atrial fibrillation; CHD, coronary heart disease; TIA, transient ischemic attack; TC, total cholesterol; LDL-C, low density lipoprotein cholesterol; TG, triglyceride; HDL-C, high density lipoprotein cholesterol; DAPT, dual antiplatelet therapy; ASA, acetylsalicacid; Clo, clopidogrel; HR MRI, high resolution magnetic resonance imaging; IPH, intraplaque hemorrhage.

**Table 2:** Location of infarction.

**Infarction location**	**Left**	**Right**	**Bilateral**
Medulla	4	9	2
Pontine	15	18	8
Midbrain	1	2	1
Cortex	5	5	0
Thalamus	1	0	0
Cerebellum	6	5	3
Corpus callosum	0	2	2

For the symptomatic patients, 25.0, 34.2, 48.7, 14.5, and 2.6% of them had RFC, lipid core, IPH, enhancement, and calcification, respectively. For the asymptomatic patients, 10.4, 37.3, 20.9, 22.4 and 9.0% of them had RFC, lipid core, IPH, enhancement, and calcification, respectively (Table 1).

**Figure 2:**
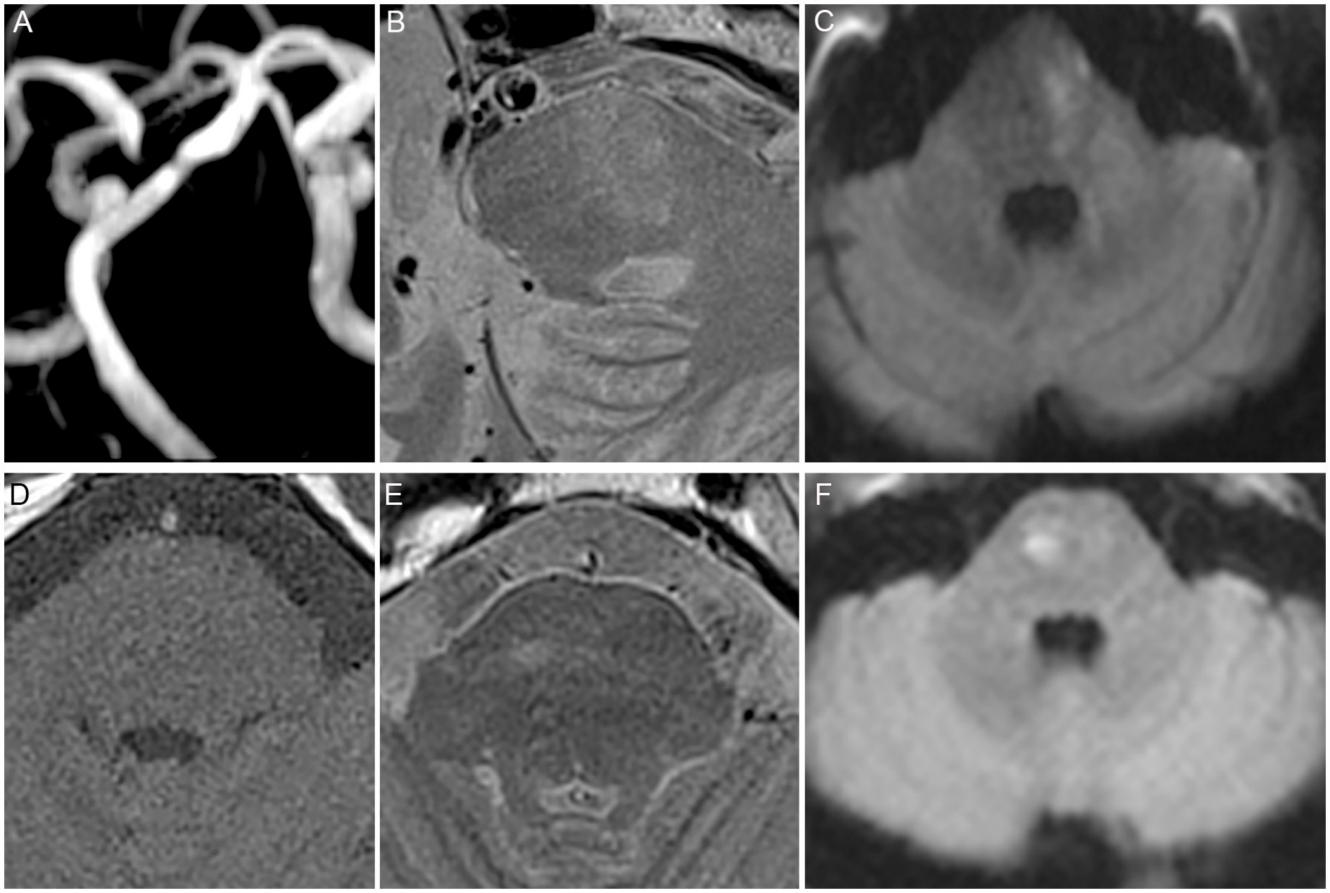
High risk features of the basilar artery (BA). **(A)** Significant BA stenosis. **(B)** Ruptured fibrous cap (RFC). **(C)** Acute infarction identified by diffusion-weighted imaging (DWI) located in the pons. **(D)** T1 and. **(E)** T2 showed abnormal signal. **(F)** Acute infarction identified by DWI located in the pons.

The use of hypoglycemic drugs, statin, RFC (Figure 2), and IPH was significantly different between asymptomatic and symptomatic patients (*P* < 0.05, Tables 1, 3). Multivariate logistic regression analysis showed that statin (8.103, 95%CI 1.476 to 44.469, *p* = 0.016, Table 4), RFC (3.157, 95%CI 1.062 to 9.382, *p* = 0.039, Table 4), and IPH (2.78, 95%CI 1.127 to 6.505, *P* = 0.026, Table 4) were independent risk factors for symptomatic patients (with acute infarction).

**Table 3:** Univariate logistic regression.

**Characteristics**	**Infarction**	**No infarction**	***P***	**Exp (B)**	**95% CI**	
	***N* = 76**	***N* = 67**			
Age (yrs)	56.5 ± 11.7	57.2 ± 13.8	0.751	0996	0.968	1.023
Male	57 (75.0%)	48 (71.6%)	0.650	1.187	0.565	2.496
Medical History						
Hypertension	42 (55.3%)	37 (55.2%)	0.964	1.012	0.597	1.716
New diagnosed	36 (47.4%)	32 (47.8%)				
Hypertension history	6 (7.9%)	5 (7.5%)				
Diabetes	24 (31.6%)	12 (17.9%)	0.069	1.811	0.955	3.435
New diagnosed	19 (25.0%)	10 (14.9%)				
Diabetes history	5 (6.6%)	2 (3.0%)				
AF	2 (2.6%)	1 (1.5%)	0.640	1.784	0.158	20.126
CHD	1 (1.3%)	1 (1.5%)	0.928	0.880	0.054	14.348
Previous stroke or TIA	8 (10.5%)	6 (9.0%)	0.753	1.196	0.393	3.642
Tumor history	4 (5.3%)	4 (6.0%)	0.854	0.875	0.210	3.644
Radiotherapy history	2 (2.6%)	1 (1.5%)	0.640	1.784	0.158	20.126
TC (mM)	4.23 ± 1.21	4.35 ± 1.18	0.571	1.091	0.808	1.472
LDL-C (mM)	2.89 ± 0.96	2.84 ± 1.09	0.813	1.043	0.736	1.478
HDL-C (mM)	1.03 ± 0.37	1.02 ± 0.25	0.785	1.164	0.392	3.460
TG (mM)	1.99 ± 1.06	1.86 ± 0.87	0.459	1.149	0.796	1.657
Smoker	30 (39.5%)	20 (29.9%)	0.344	1.291	0761	2.192
Current smoker	24 (31.6%)	15 (22.4%)				
Previous smoker	6 (7.9%)	5 (7.5%)				
Drinker	15 (19.7%)	16 (23.9%)	0.915	1.035	0.553	1.936
Current drinker	13 (17.1%)	12 (17.9%)				
Previous drinker	2 (2.6%)	4 (6.0%)				
Medication						
Antihypertensive	35 (46.1%)	28 (41.8%)	0.823	1.189	0.613	2.307
Hypoglycemic	24 (31.6%)	11 (16.4%)	0.038	2.350	1.048	5.268
Statin	74 (97.4%)	59 (88.1%)	0.046	5.017	1.026	24.523
Antiplatelet	76 (100.0%)	67 (100.0%)	0.402	1.197	0.786	1.823
DAPT	49 (64.4%)	43 (64.2%)				
ASA or Clo	27 (35.5%)	24 (35.8%)				
Anticoagulation	1 (1.3%)	2 (3.0%)	0.499	0.433	0.038	4.889
HR MRI						
Ruptured fibrous cap	19 (25.0%)	7 (10.4%)	0.028	2.857	1.117	7.309
Lipid core	26 (34.2%)	25 (37.3%)	0.699	0.874	0.440	1.734
IPH	37 (48.7%)	17 (25.4%)	0.005	2.790	1.371	5.680
Enhancement	11 (14.5%)	15 (22.4%)	0.224	0.587	0.248	1.385
Calcification	2 (2.6%)	6 (9.0%)	0.122	0.275	0.054	1.411

AF, atrial fibrillation; CHD, coronary heart disease; TIA, transient ischemic attack; TC, total cholesterol; LDL-C, low density lipoprotein cholesterol; TG, triglyceride; HDL-C, high density lipoprotein cholesterol; DAPT, dual antiplatelet therapy; ASA, acetylsalicacid; Clo, clopidogrel; HR MRI, high resolution magnetic resonance imaging; IPH, intraplaque hemorrhage.

**Table 4:** Multivariate logistic regression.

**Characteristics**	**B**	**S.E**.	**Wald**	**P**	**Exp (B)**	**95% CI**	
Diabetes	−0.248	0.84	0.087	0.768	0.781	0.151	4.046
Hypoglycemic	1.271	1.093	1.35	0.245	3.563	0.418	30.378
Statin	2.092	0.869	5.8	0.016	8.103	1.476	44.469
Plaque characteristics							
Ruptured fibrous cap	1.150	0.556	4.280	0.039	3.157	1.062	9.382
Lipid core	0.134	0.445	0.091	0.764	1.143	0.478	2.737
IPH	0.996	0.447	4.964	0.026	2.708	1.127	6.505
Enhancement	−1.072	0.546	3.858	0.05	0.342	0.117	0.998
Calcification	−1.092	0.887	1.515	0.218	0.336	0.059	1.910

## Discussion

The present study showed that the RFC and IPH of BA plaque were independent risk factors for symptomatic patients. It was the first study to report that the RFC of BA plaques was associated with acute infarction. The results suggested that HR-MRI could be used to identify the high-risk BA plaques, which were the potential targets of further intervention.

A ruptured fibrous cap is considered as the risk factor of the vascular event because, in this circumstance, the platelets could adhere to the collagen through the non-intact endothelial cells. In the extracranial artery, RFC of carotid plaques was reported to be associated with the onset of stroke (10). For the intracranial artery, the present study was the first one to report the relationship between RFC in BA plaques and acute infarction as it was hard to detect RFC in the middle cerebral artery (MCA) (6). As for the BA, the larger diameter and the relatively straight shape provided the opportunity for HR-MRI to detect RFC (11). Moreover, BA has more cisternal space when compared with the MCA (7). The rupture of the fibrous cap is dependent on endogenous factors, such as its thickness, and exogenous factors, such as the stress on the plaque. The rupture was likely to occur when the mechanical stress exceeded the material strength of the fibrous cap (12).

Intraplaque hemorrhage of BA plaques was another risk factor for acute infarction. Our data were consistent with others (7, 13, 14). Irrespective of the size of the plaques, IPH was a high-risk factor for the onset of stroke; moreover, the size of IPH was positively related to the onset of stroke (15). However, some studies showed no clinical significance of IPH of BA plaque (8). This may be due to the different cohorts involved in the study. The occurrence of IPH was attributed to fragile neovascularity with endothelial disruption, which caused a hyperintense signal in the T1-weighted images (16).

Contrast enhancement was a vital feature of the intracranial atherosclerotic plaque on HR-MRI. Xu et al. showed that plaque enhancement in the MCA plaque was related to the onset of stroke (17). As for BA, Wang et al. showed that plaque enhancement was a risk factor for the onset of stroke (8). Our data showed no significant difference (*p* = 0.05) in the multivariate regression analysis.

The previous study also suggested that the lipid core of BA plaque was also related to acute stroke (18). However, our data showed no significant difference between symptomatic and asymptomatic patients. Lee et al. showed that this inconsistency may be due to the small size of the sample (only 10 patients were recruited) (18). In the coronary artery, calcification was the feature of non-vulnerable plaque (19). Our study reported a non-significant negative relationship between calcification and the onset of stroke using the multivariable regression analysis.

There were some limitations to this study. The first is a relatively small sample size included in the present study although it was the largest cohort of BA plaques assessed by HR-MRI. The second is the resolution of HR-MRI which was not satisfactory for some patients. Some case series studies used optical coherence tomography (OCT) to visualize the detailed features of BA plaques such as thin fibrous cap and fibrous erosion. The advantage of HR-MRI compared with OCT was non-invasive. However, HR-MRI for the detection of intracranial plaque lacks histopathological correlation. The third limitation is that an acute infarction was determined by DWI while some strokes cannot be detected by DWI images, especially in the brainstem, which may cause some bias.

In conclusion, RFC and IPH of BA plaques on HR-MRI were independent risk factors for the new onset of stroke. This study provided a non-invasive method to predict the onset of stroke. Further studies involving larger samples should be conducted.

## Data availability statement

The original contributions presented in the study are included in the article/supplementary material, further inquiries can be directed to the corresponding authors.

## Ethics statement

The studies involving human participants were reviewed and approved by the Ethics Committees of Guangzhou Medical University, Longhua Hospital, Sun Yat-sen University, the First Affiliated Hospital of Soochow University, Wuxi People's Hospital and Shanghai General Hospital, according to the principles expressed in the Declaration of Helsinki. The patients/participants provided their written informed consent to participate in this study.

## Author contributions

SL and JW: data acquisition and writing original draft preparation. RH: data curation. CL and HC: image review and visualization. ZQ: validation and funding acquisition. YJ: data curation, validation, and funding acquisition. LW: conceptualization, supervision, writing review, editing and funding acquisition. All authors contributed to the article and approved the submitted version.

## Funding

This study was financially supported by the National Natural Science Foundation of China (81501009), the Guangzhou Science and Technology Project (202201020393), and the Guangdong Provincial Medical Science and Technology Research Fund (A2021406) to LW; the National Natural Science Foundation of China (81870933), GuangDong Basic and Applied Basic Research Foundation (2021A1515012351), and the Opening Lab Program of Guangzhou Medical University (0506308) to YJ; Special Funds for the Cultivation of Guangdong College Students' Scientific and Technological Innovation (Climbing Program Special Funds pdjh2020b0492) to RH; and the Scientific Program of Guangzhou Municipal Health Commission (20191A011083) to ZQ. The funding sources had no involvement in the design of the study and collection, analysis, interpretation of data, and writing of the manuscript.

## Conflict of interest

The authors declare that the research was conducted in the absence of any commercial or financial relationships that could be construed as a potential conflict of interest.

## Publisher's note

All claims expressed in this article are solely those of the authors and do not necessarily represent those of their affiliated organizations, or those of the publisher, the editors and the reviewers. Any product that may be evaluated in this article, or claim that may be made by its manufacturer, is not guaranteed or endorsed by the publisher.
